# H5N1 Avian Influenza, Kampot Province, Cambodia

**DOI:** 10.3201/eid1201.050914

**Published:** 2006-01

**Authors:** Shaun K. Morris

**Affiliations:** * University of Toronto, Toronto, Ontario, Canada

**Keywords:** avian influenza, Cambodia, disease outbreaks

**To the Editor:** As a resident in pediatrics with an interest in infectious diseases, I was fascinated by the range and scope of conditions I saw in May and June of 2005 in local children in Kampot Province, a mainly rural area in southeastern Cambodia. This province, near the Vietnamese border, is the epicenter of H5N1 avian influenza in Cambodia, with all 4 known human cases of the disease. All of these documented human cases have been fatal.

While the World Health Organization and the Cambodian Ministry of Health have engaged in public education, the village-level response to a pathogen of potential global importance is evolving. As part of my work in Cambodia, I made numerous information-gathering visits to villages in Kampot Province. Though village elders and health workers had often heard of the "bird flu," most of the villagers I spoke to had not. Many persons did not know whom to contact should their chickens or other birds die. Those who knew about H5N1 influenza told me that, without adequate compensation for culling flocks, little incentive would exist to report bird deaths. In a typical village, chickens, ducks, and pigs intermingled with each other and with humans underneath or around homes on stilts. General knowledge of infection control practices among villagers was minimal.

The dissemination of information into a rural, agricultural society such as that in the southeast of Cambodia is a difficult task. Many rural inhabitants do not have televisions or radios and may infrequently travel to larger towns. Health workers from international groups, nongovernmental organizations, and the government are often required to travel on foot or motorbike through fields and forests to reach and educate the population. Government health workers lack the personnel and resources to adequately identify and investigate potential cases, and Cambodia has substantially fewer microbiology laboratories than do neighboring Thailand and Vietnam.

Should a pandemic of avian influenza occur, it will almost certainly originate in Southeast Asia. Cambodian and international health organizations have recognized the country’s potential key role in propagation of an impending pandemic agent. However, because of its history and current economic state, Cambodia is less able to respond to the avian influenza threat than its neighbors. In recognition of this fact, the World Health Organization and the Cambodian Ministry of Health have stated that the prevention, control, and identification of avian influenza are national priorities. Additionally, international funds have been flowing into Cambodia to assist with avian H5N1 influenza surveillance and case investigation. Much work remains to be done; we hope that by combining international resources and policy with domestic expertise and effort, Cambodia will mount a successful response against this emerging threat.

